# Construction of the miRNA-mRNA Regulatory Networks and Explore Their Role in the Development of Lung Squamous Cell Carcinoma

**DOI:** 10.3389/fmolb.2022.888020

**Published:** 2022-05-31

**Authors:** Xingchen Fan, Xuan Zou, Cheng Liu, Jiawen Liu, Shuang Peng, Shiyu Zhang, Xin Zhou, Tongshan Wang, Xiangnan Geng, Guoxin Song, Wei Zhu

**Affiliations:** ^1^ Department of Oncology, First Affiliated Hospital of Nanjing Medical University, Nanjing, China; ^2^ First Clinical College of Nanjing Medical University, Nanjing, China; ^3^ Department of Gastroenterology, First Affiliated Hospital of Nanjing Medical University, Nanjing, China; ^4^ Department of Clinical Engineer, First Affiliated Hospital of Nanjing Medical University, Nanjing, China; ^5^ Department of Pathology, First Affiliated Hospital of Nanjing Medical University, Nanjing, China

**Keywords:** miRNA, MiRNA-mRNA networks, lung squamous cell carcinoma, TCGA, GEO, PCR

## Abstract

**Purpose:** MicroRNA (miRNA) binds to target mRNA and inhibit post-transcriptional gene expression. It plays an essential role in regulating gene expression, cell cycle, and biological development. This study aims to identify potential miRNA-mRNA regulatory networks that contribute to the pathogenesis of lung squamous cell carcinoma (LUSC).

**Patients and Methods:** MiRNA microarray and RNA-Seq datasets were obtained from the gene expression omnibus (GEO) databases, the cancer genome atlas (TCGA), miRcancer, and dbDEMC. The GEO2R tool, “limma” and “DEseq” R packages were used to perform differential expression analysis. Gene enrichment analysis was conducted using the DAVID, DIANA, and Hiplot tools. The miRNA-mRNA regulatory networks were screened from the experimentally validated miRNA-target interactions databases (miRTarBase and TarBase). External validation was carried out in 30 pairs of LUSC tissues by Real-Time Quantitative Reverse Transcription PCR (qRT-PCR). Receiver operating characteristic curve (ROC) and decision curve analysis (DCA) were conducted to evaluate the diagnostic value. Clinical, survival and phenotypic analysis of miRNA-mRNA regulatory networks were further explored.

**Results:** We screened 5 miRNA and 10 mRNA expression datasets from GEO and identified 7 DE-miRNAs and 270 DE-mRNAs. After databases screening and correlation analysis, four pairs of miRNA-mRNA regulatory networks were screened out. The miRNA-mRNA network of miR-205-5p (up) and PTPRM (down) was validated in 30 pairs of LUSC tissues. MiR-205-5p and PTPRM have good diagnostic efficacy and are expressed differently in different clinical features and are related to tumor immunity.

**Conclusion:** The research identified a potential miRNA-mRNA regulatory network, providing a new way to explore the genesis and development of LUSC.

## Introduction

Lung cancer is the leading cause of cancer death worldwide, accounting for approximately 18% of all cancer deaths (Bray et al., 2018). Lung cancer has the highest morbidity and mortality rate in China, accounting for 24.7% and 29.4%, respectively. There are four main histological types of lung cancer: adenocarcinoma, squamous cell carcinoma, small cell carcinoma, and large cell carcinoma, of which lung squamous cell carcinoma (LUSC) is one of the major histological subtypes (Travis et al., 2015). Survival rates for lung squamous cell carcinoma remain low, with 5-year survival rates of only 10–20% in most parts of the world. The diagnostic stage is a significant determinant of the prognosis of lung squamous cell carcinoma, and the detection of molecular markers for early diagnosis, prognosis, and therapeutic targets of LUSC has become very urgent (Allemani et al., 2015).

MicroRNA (miRNA) is a kind of single-stranded, non-coding small RNA molecule (containing about 22 nucleotides) found in plants, animals, and viruses. miRNA binds target mRNA to inhibit post-transcriptional gene expression and plays an essential role in regulating gene expression, cell cycle, and the timing of biological development (Bartel 2009). The expression patterns of miRNAs have been reported to have the potential to identify various types of cancers (Slattery et al., 2016; Liu et al., 2018; Zhang et al., 2019). Even though numerous studies have been conducted on miRNA expression and function in LUSC, systematic and comprehensive analysis of the miRNA-mRNA regulatory network based on clinical samples from LUSC are still lacking. The construction of a potential miRNA-mRNA regulatory network will help reveal the relatively comprehensive molecular mechanism of LUSC.

Here, we downloaded data from the GEO, TCGA-LUSC, miRcancer, and dbDEMC databases to screen for differential miRNAs (De-miRNAs) and mRNA (DE-mRNAs) between LUSC and normal tissue. The interactions between DE-miRNAs and DE-mRNAs were determined using Tarbase and miRTarbase databases, which are experimentally validated miRNA-target interaction databases. We further validated the DE-miRNAs and DE-mRNAs in 30 pairs of LUSC tissues by qRT-PCR. In summary, the interaction of the miRNA-mRNA regulatory networks have been researched in detail, providing new ideas or strategies for further development and application in clinical setting, for early diagnosis and better treatment.

## Materials and Methods

### Data Acquisition and Processing of miRNA and Gene Expression Profiles

**FIGURE 1:**
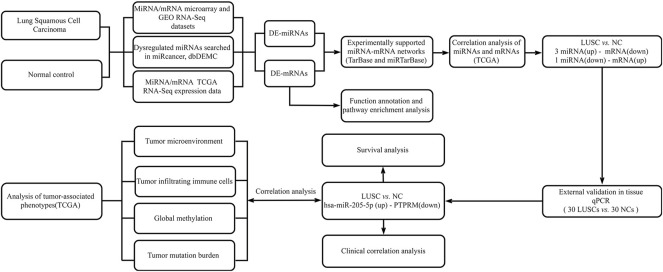
Flow chart for identifying the miRNA-mRNA regulatory pairs and the comprehensive analysis of regulatory pairs role in lung squamous cell carcinoma (LUSC).

We downloaded the miRNA and mRNA sequencing expression profiles and associated clinicopathological data of TCGA-LUSC from the GDC data portal at the National Cancer Institute (https://portal.gdc.cancer.gov/). We searched lung squamous cell carcinoma relevant gene microarray expression datasets from the Gene Expression Omnibus (GEO) database (http://www.ncbi.nlm.nih.gov/geo/) with the following keywords: “lung squamous cell carcinoma”. Filters were set to “series” and “Expression profiling by array”, “Non-coding RNA profiling by array” and “*Homo sapiens*”. The inclusive criteria of datasets is that the datasets have miRNA or mRNA expression values in lung squamous cell carcinoma tumors and normal tissues. Then we screened 5 eligible miRNA datasets and 10 eligible mRNA datasets. First, we screened differentially expressed miRNA (DE-miRNAs) or mRNA (DE-mRNAs) in each dataset. The DE-miRNAs and DE-mRNAs were obtained from microarray expression profiles using the web analysis tool GEO2R, which is used to compare groups of samples by the GEOquery and limma R packages from the Bioconductor project in the GEO database (http://www.ncbi.nlm.nih.gov/geo/geo2r/). The cut-off criteria were *p*-value < 0.05 and |log2 (fold change) | ≥ 1. Then, we summarized the differential miRNAs or mRNAs screened out from each dataset. We also collected differentially expressed miRNAs from miRCancer and Database of Differentially Expressed MiRNAs in human Cancers (dbDEMC) (Xie et al., 2013; Yang et al., 2017). An overview of the workflow steps is shown in Figure 1.

### Identification and Function Analysis of miRNA-mRNA Networks

TarBase (Karagkouni et al., 2018) (http://www.microrna.gr/tarbase) is a reference database, specifically for index experiments support the miRNA targets. It integrates information on cell-type specific miRNA–gene regulation, while hundreds of thousands of miRNA-binding locations are reported. miRTarBase (Chou et al., 2018) is a comprehensive annotated and experimentally validated miRNA-target interaction database for miRNA-related researches. Tarbase and miRTarBase are used to construct the miRNA-mRNA regulatory networks. We analyzed the correlation between miRNA and mRNA in TCGA-LUSC, and screened the miRNA-mRNA regulatory networks according to the results. The online program DAVID (http://david.abcc.ncifcrf.gov/), a database for annotation, visualization and integrated discovery, is a comprehensive tool for researchers and scholars to understand the biological meaning behind multiple genes. We used DAVID, DIANA-MirPath (Vlachos et al., 2015), a miRNA pathway analysis web-server, and Hiplot (https://hiplot.com.cn), a comprehensive web platform for scientific data visualization, to perform Gene Ontology (GO) functional analysis and Kyoto Encyclopedia of Genes Genomes (KEGG) pathways analysis. Single sample gene set Enrichment analysis (ssGSEA) is an extension of the GSEA method that allows the definition of an enrichment score that represents the absolute degree of enrichment of the gene set in each sample within a given dataset (Hoadley et al., 2018; Xiao et al., 2020). The ssGSEA data of TCGA-LUSC were downloaded from UCSC Xena (https://xena.ucsc.edu/) to analyze the possible enrichment pathways of DE-miRNAs and DE-mRNAs.

### Sample Collection and RNA Isolation

FFPE specimens were obtained from LUSC patients undergoing radical surgery in the First Affiliated Hospital of Nanjing Medical University. All samples were collected with the written informed consent from the patient and the prior approval of the Institutional Review Boards of the First Affiliated Hospital of Nanjing Medical University (ID: 2016-SRFA-148) according to the Declaration of Helsinki. The clinical features of the 30 LUSC patients are shown in Table 1. Total RNA was extracted from FFPE specimens using the TIANGEN RNAprep Pure FFPE kit (Tiangen, Beijing, China). The acquired RNA from each sample was lysed in 40 µl RNase-free water and stored at −80°C until use. The concentration and purity of RNA were analyzed by the Nanodrop 2000 spectrophotometer (NanoDrop Technologies, Wilmington, DE, United States ) (A260/A280 = 1.8–2, A260/A230 > 1.7).

**TABLE 1:** Clinicopathological and molecular features of LUSC patients.

Variables	Number of cases	Rate (%)
	(*n* = 30)	
Age (years)		
≤60	8	27
>60	22	73
Gender		
Female	2	6.7
Male	28	93.3
Tumor size (cm)		
≤3	19	63.3
>3	11	36.7
TNM stage		
I-II	20	66.7
III-IV	10	33.3
Lymph node metastasis		
No	16	53.3
Yes	14	46.7
Bronchial invasion		
No	20	66.7
Yes	10	33.3
Pathological grading		
I-II	7	23.3
III	23	76.7

### Quantitative Reverse Transcription PCR (qRT-PCR) Assay

According to the manufacturer’s instructions, the external validation was verified by qRT-PCR using PrimeScript RT reagent Kit (Takara) and SYBR Premix Ex Taq II (Takara) after adding a poly (A) tail to RNA by Poly (A) Polymerase Kit (Takara). The sequences of PCR primers are listed in Supplementary Table S1. This process was run on qTOWER³ 84 (Analytik Jena) at 95°C for 20 s, followed by 40 cycles of 10 s at 95°C, 20 s at 60°C. The expression levels of miRNAs and mRNAs in tissue were calculated using the 2^−ΔΔCt^ method (Livak and Schmittgen 2001). (U6 as endogenous reference miRNA for sample normalization; 18s rRNA as endogenous reference mRNA for sample normalization; ΔCt = Ct_miRNA/mRNA_− Ct_normalizer_; Ct: the threshold cycle).

### Evaluation of Interactions of miRNA-mRNA Networks and Tumor-Relative Phenotypes

We downloaded the infiltrating immune cell types data from the TCGA website and calculated using CIBERSORT (https://cibersort.stanford.edu/index.php/), a general calculation method used to quantify cell fractions from a large number of tissue gene expression profiles (GEP). Combining support vector regression with prior knowledge of the expression profile of purified white blood cell subsets, CIBERSORT was able to accurately estimate the immune component of tumor biopsy (Chen et al., 2018). The stromal and immune levels of TCGA-LUSC samples were evaluated using ESTIMATE (Estimation of STromal and Immune cells in MAlignant Tumour tissues using Expression data) software. This method uses gene expression signatures to infer the fraction of stromal and immune cells in tumour samples (Yoshihara et al., 2013). We used the UCSC Xena platform (https://xena.ucsc.edu/) to obtain the methylation level of CpG sites in TCGA-LUSC samples, which is detected by the Illumina Infinium HumanMethylation450 BeadChips platform. The amount of somatic variants per megabase (MB) of the genome was measured by tumor mutation burden (TMB).

### Statistical Analysis

We used GraphPad Prism software, IBM SPSS Statistics v.26 software (IBM Corporation, Armonk, NY, United States ), and R language v3.6.3 (https://cran.r-project.org/) for data analysis. The *t*-test was performed to analyze DE-miRNAs and DE-mRNAs. The statistically significant criteria of DE-miRNAs and DE-mRNAs are |log2FC| >0.58 and *p* < 0.05. The area under the ROC curve (AUC) and decision curve analysis (DCA) based on logistic regression were used to evaluate the diagnostic efficacy of miRNA-mRNA networks. The Pearson correlation method was used to calculate the correlation between DE-mRNAs or DE-miRNAs and tumor-related phenotypes. Prognostic analysis was performed using the “survival” package.

## Results

### Identification of Differentially Expressed miRNAs and mRNAs in LUSC

**FIGURE 2:**
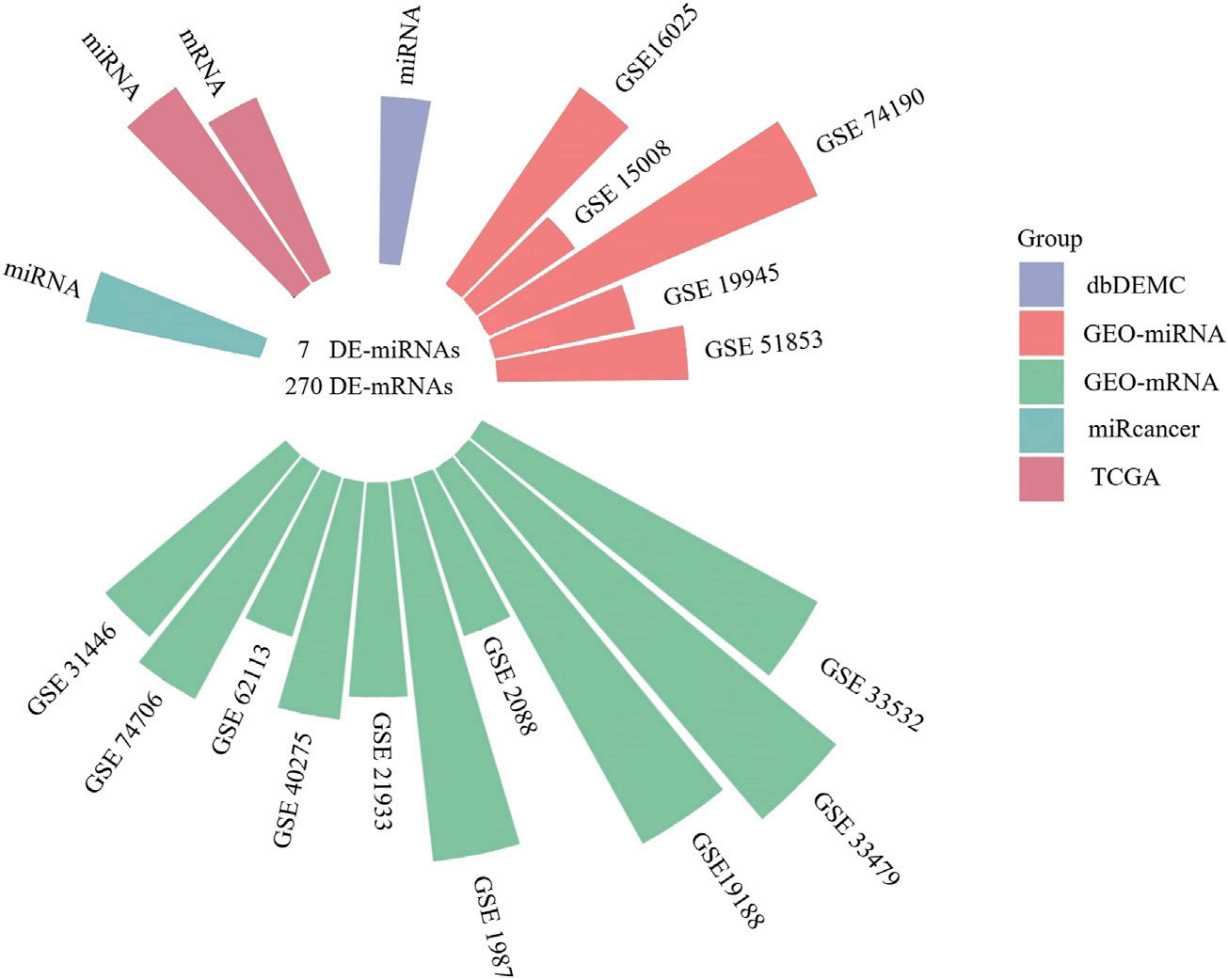
The circular bar chart showing the datasets from different sources for screening differentially expressed miRNAs and mRNAs.

We screened 5 miRNA and 10 mRNA expression datasets from GEO, and the information of 15 GEO datasets is shown in Table 2. The log2 fold change (LUSC vs. NC) was used to screen the DE-mRNAs and DE-miRNAs. As shown in Figure 2, the 7 DE-miRNAs and 270 DE-mRNAs were the intersections of the TCGA, GEO, dbDEMC, and miRcancer databases. The results of the preliminary screening are presented in Supplementary Table S2. We utilized DIANA-MirPath to predict the possible functions of the 7 DE-miRNAs, as well as the targets of DE-miRNAs enriched in Fatty acid biosynthesis, Cell cycle, Fatty acid metabolism etc,. (Supplementary Figure S1A). Then, we also analyzed the 270 DE-mRNAs using cluster profile on the Hiplot platform. KEGG pathway enrichment analysis revealed that the dysregulated DE-mRNAs were enriched in the cell cycle, DNA replication, p53 signaling pathway etc,. (Supplementary Figure S1C). The top 10 GO terms of the DE-miRNAs and DE-mRNAs were presented in Supplementary Figures S1B,D, including cellular component, extracellular exosome and extracellular space in the CC category, DNA metabolic process, DNA replication and cell division in the BP category, protein binding transcription factor activity, protein binding, and ATP binding in the MF category.

**TABLE 2:** Information pertaining to the selected GEO datasets for LUSC.

	Experiment type	Source name	GEO Accession	Platform	Group	
					Tumor	Control
microRNA expression	Array	Tissue	GSE16025	GPL5106	61	10
			GSE15008	GPL8176	116	116
			GSE74190	GPL19622	30	44
			GSE19945	GPL9948	5	8
			GSE51853	GPL7341	29	5
mRNA expression	Array	Tissue	GSE33532	GPL570	16	20
			GSE33479	GPL6480	14	27
			GSE19188	GPL570	27	65
			GSE2088	GPL962	48	30
			GSE1987	GPL91	16	7
			GSE21933	GPL6254	10	21
			GSE40275	GPL15974	4	43
			GSE62113	GPL14951	7	9
			GSE74706	GPL13497	8	18
			GSE31446	GPL9244	18	18

### Screening of miRNA-RNA Regulatory Networks Associated With LUSC

**FIGURE 3:**
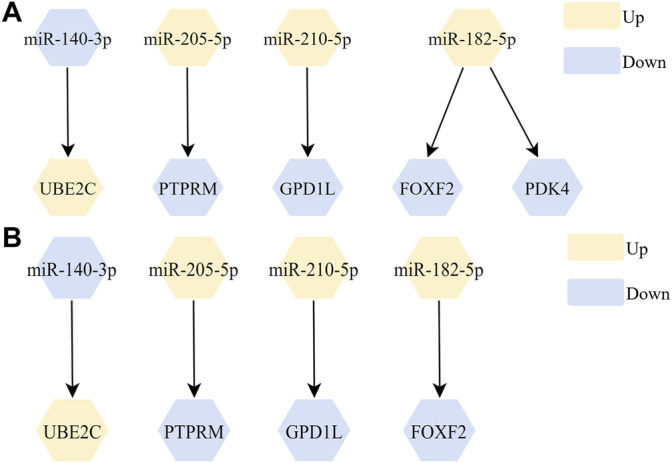
The screened miRNA-mRNA regulation networks. **(A)** 5 miRNA-mRNA regulatory networks were filtered out after screening from miRtarbase and Tarbase databases; **(B)** 4 miRNA-mRNA regulatory networks were filtered out after correlation analysis.

We further screened miRtarbase and Tarbase database to establish potential miRNA-mRNA regulatory networks, and screened out 5 miRNA-mRNA regulatory networks (Figure 3A). The complete prediction network of miR-205-5p is shown in Supplementary Table S3. Then, we filtered out 4 miRNA-mRNA networks with significant correlation (adjusted *p*-value < 0.05) in TCGA-LUSC listed in Figure 3B and full statistical results listed in Supplementary Table S4.

### Validation of the Expression of miRNAs and mRNAs in LUSC Tissue

**FIGURE 4:**
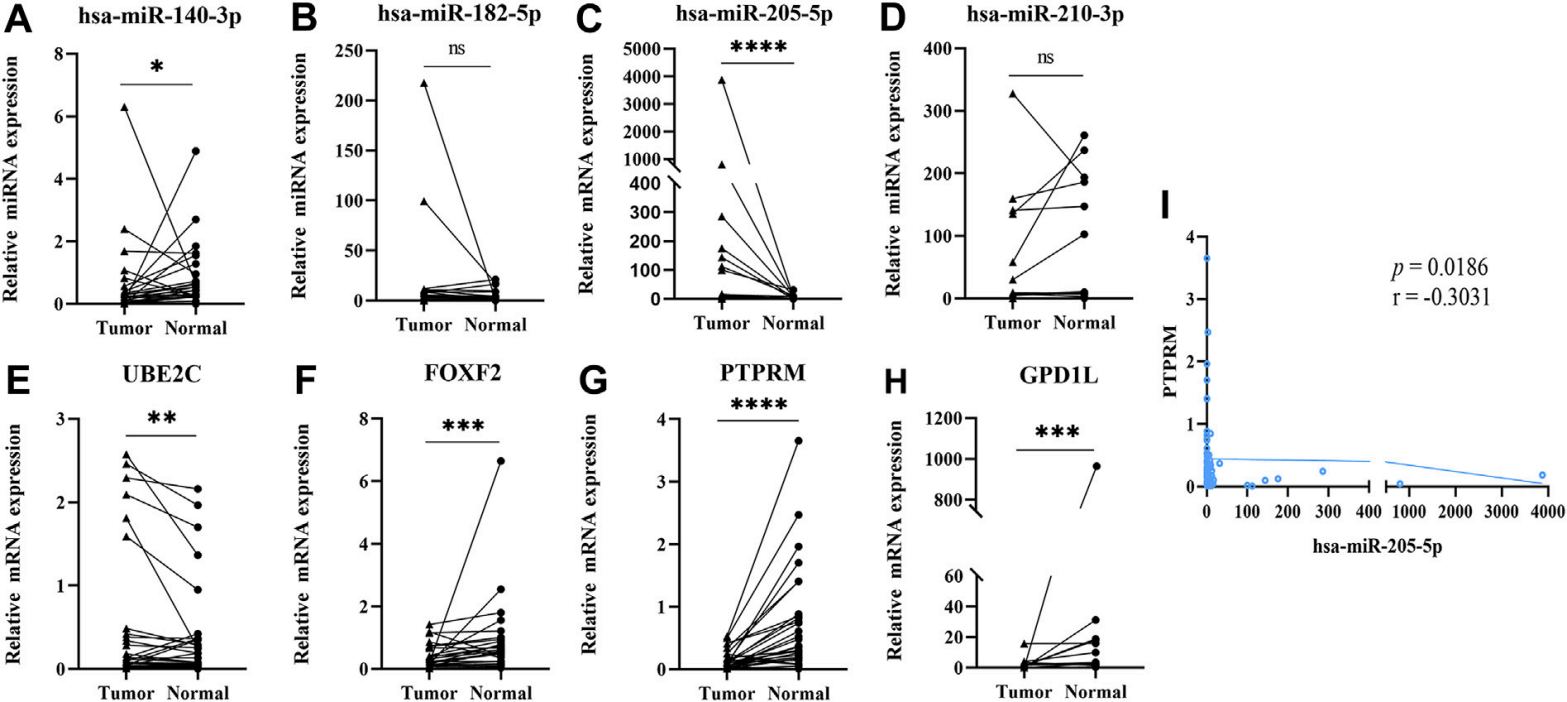
Validating the expression of 4 DE-miRNAs and 4 DE-mRNAs by RT-qPCR (Data are presented as mean ± SEM; *p < 0.05, **p < 0.01, ***p < 0.001, ****p < 0.0001). **(A)** miR-140-3p; **(B)** miR-182-5p; **(C)** miR-205-5p; **(D)** miR-210-3p; **(E)** UBE2C; **(F)** FOXF2; **(G)** PTPRM; **(H)** GPD1L; **(I)** Pearson’s correlation analysis of miR-205-5p and PTPRM.

In order to prove the differential expression of the DE-miRNAs and DE-mRNAs, we further validated them in 30 pairs of matched tumors and adjacent normal tissues by qRT-PCR. As shown in Figure 4, the expression of miR-205-5p (*p* < 0.0001) and UBE2C (*p* = 0.0093) were upregulated in tumor tissues, while the expressions of miR-140-3p (*p* = 0.0293), PTPRM (*p* < 0.0001), GPD1L (*p* = 0.0002), and FOXF2 (*p* < 0.0001) were downregulated in tumor tissues. At the same time, miR-182-5p (*p* = 0.2286) and miR-210-3p (*p* = 0.0879) showed no significant difference in tumor tissues compared with normal tissues. Spearman correlation analysis of the interaction between DE-miRNAs and DE-mRNAs, showed that miR-205-5p was significantly correlated with PTPRM (*p* = 0.0186, r = −0.3031). IHC images from the HPA database showed that PTPRM expression in LUSC was lower than in normal control shown in Supplementary Figure S2.

### Evaluation of Diagnostic Value of miRNA-mRNA Regulatory Networks in LUSC

**FIGURE 5:**
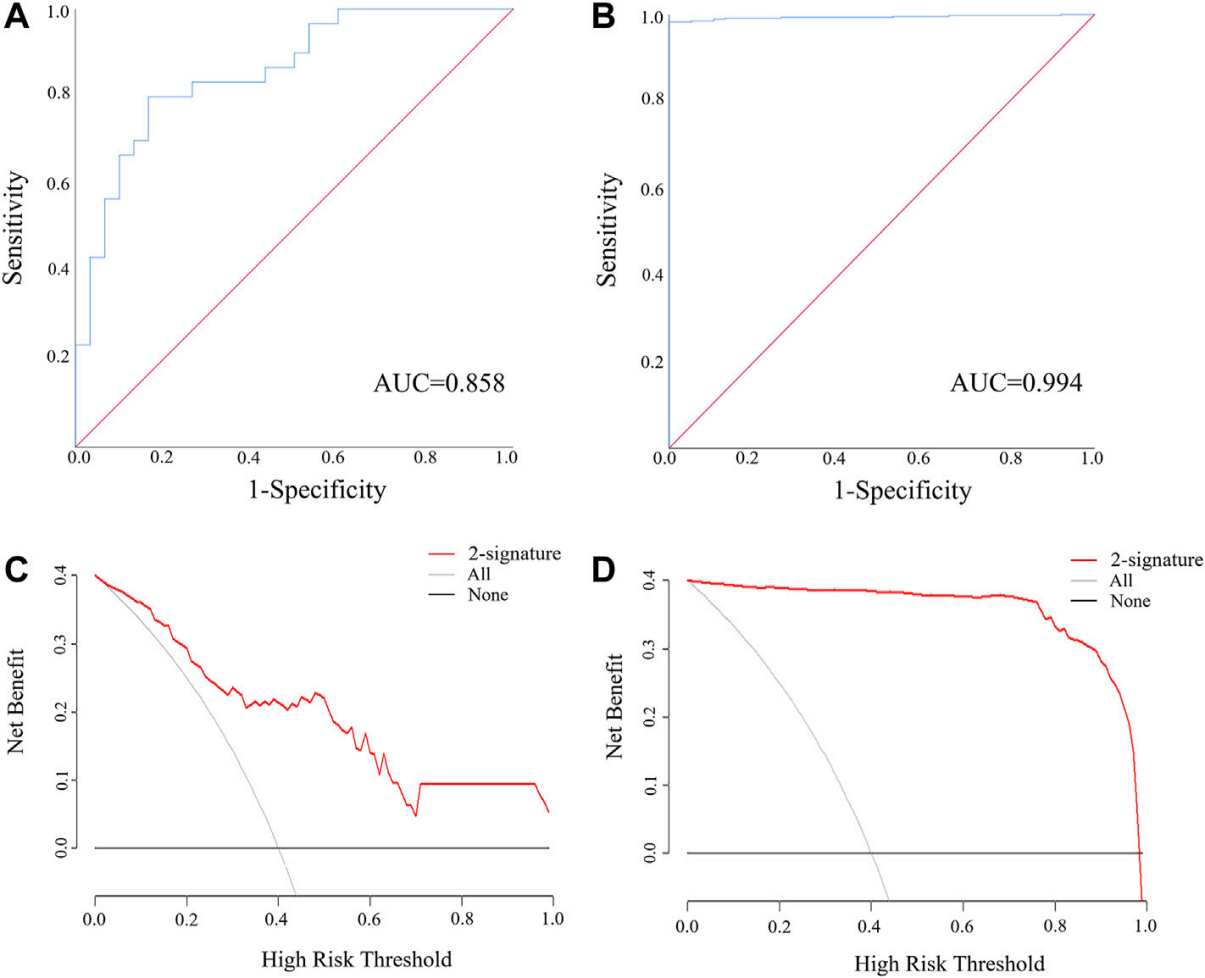
The ROC and DCA curve of miR-205-5p and PTPRM regulatory pair for discriminating LUSC patients from NCs. **(A)** The ROC of miR-205-5p and PTPRM regulatory pair in the external validation (AUC = 0.858, 95% CI: 0.746–0.951, *p* < 0.0001); **(B)** The ROC of miR-205-5p and PTPRM regulatory pair in the TCGA-LUSC (AUC = 0.994, 95% CI: 0.989–1.000, *p* < 0.0001); **(C)** The DCA of miR-205-5p and PTPRM regulatory pair in the external validation. **(D)** The DCA of miR-205-5p and PTPRM regulatory pair in the TCGA-LUSC.

MiR-205-5p and PTPRM were combined into a panel by logistic regression analysis, and the equation for calculating the probability of LUSC was: Logit(P) = 1.009 + 0.03 × miR-205-5p—4.767 × PTPRM. As is demonstrated in Figures 5A,B, the AUC of the panel was 0.994 (95% CI: 0.989–1.000, *p* < 0.0001) in TCGA-LUSC and 0.858 (95% CI: 0.746–0.951, *p* < 0.0001) in the external validation. The DCA showed that the miRNA-mRNA network had good diagnostic performance under arbitrary threshold probability (Figures 5C,D).

### Correlation Analysis of LUSC Clinical-Pathological Features and Prognosis With the Expression of miRNA and mRNA Expression Levels

Based on the analysis of FIGO stages, there was no statistically significant difference in the expression of miR-205-5p and PTPRM in the early stage (I) and advanced stage (II + III + IV) (Supplementary Figures S3A,B). The expression of miR-205-5p in male patients was higher than that in female patients (Supplementary Figures S3C,D), and PTPRM was higher in age over 68 years (Supplementary Figures S3E,F). Univariate and multivariate cox regression analyses were used to estimate the Hazard ratio (HR) of different clinical features in the TCGA-LUSC. K-M survival analysis and cox regression analysis showed that miR-205-5p and PTPRM were not associated with prognosis (Supplementary Figure S4).

### Analysis of Tumor-Related Phenotypes Associated With miRNA-mRNA Network

**FIGURE 6:**
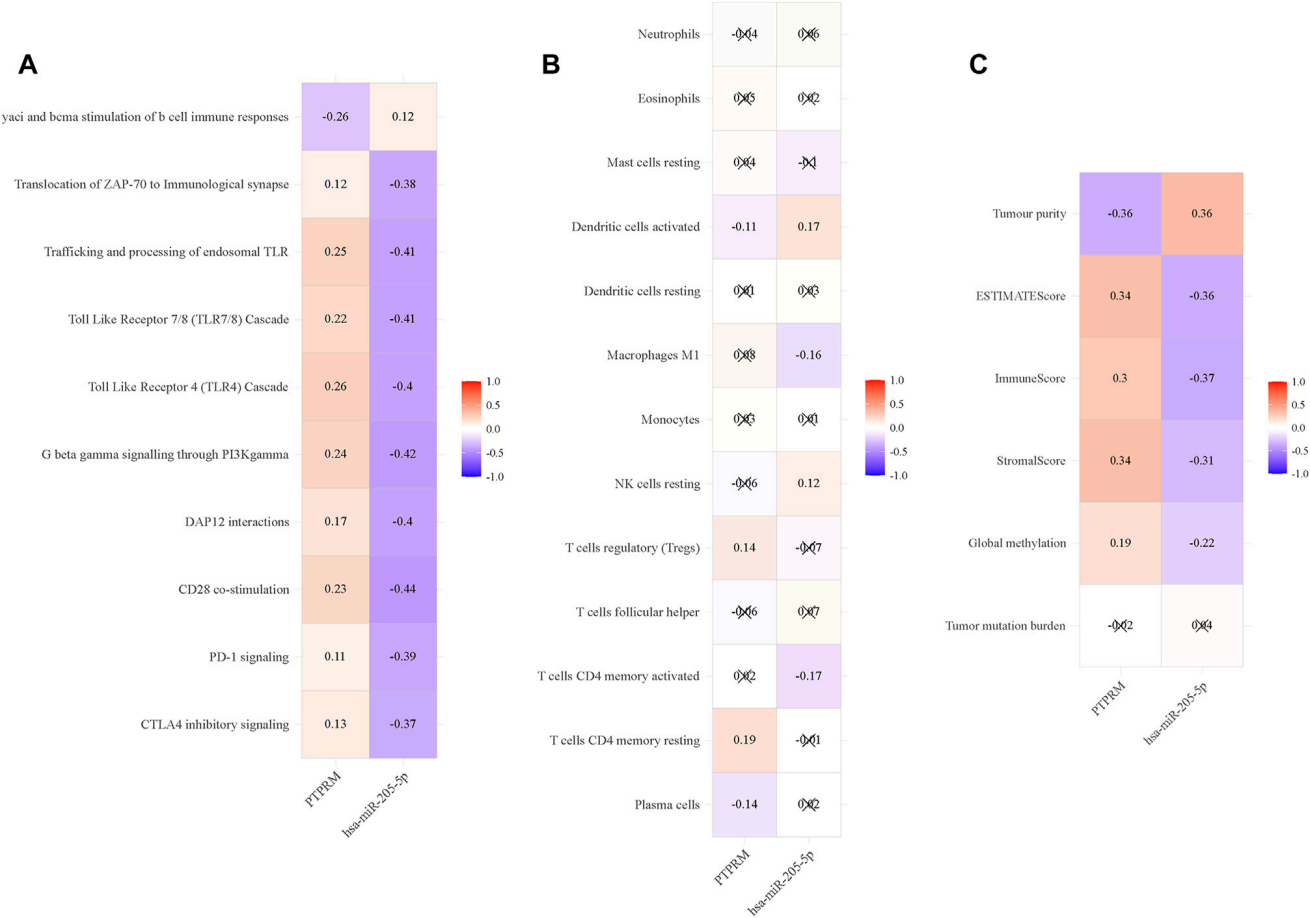
The association between immune-related phenotypes and miR-205-5p and PTPRM in TCGA-LUSC. **(A)** The correlation analysis of miR-205-5p and PTPRM and ssGSEA of TCGA-LUSC; **(B)** The correlation analysis of miR-205-5p and PTPRM and immune cells; **(C)** The correlation analysis of miR-205-5p and PTPRM and global methylation, tumor mutation burden and tumor microenvironment factors.

We downloaded the ssGSEA enrichment score of TCGA-LUSC data from UCSC Xena, and then analyzed the correlation between expression values of miR-205-5p and PTPRM and enrichment score. The results showed that miR-205-5p and PTPRM were highly correlated with immune-related pathways, such as yaci and bcma stimulation of b cell immune responses and Translocation of ZAP-70 to Immunological synapse (Figure 6A). Therefore, we further analyzed its correlation with immune cells to explore its role in tumor immunity. We conducted a differential analysis of the immune cell data in TCGA-LUSC and found that 13 types of immune cells differed between tumor and normal tissues listed in Supplementary Table S5. Then, the correlation analysis of differential immune cells with miR-205-5p and PTPRM were carried out in the following research. As shown in Figure 6B, the expression of miR-205-5p and PTPRM were correlated with dendritic cells activate. We used CIBERSORT to calculate the proportion of various immune cells in each TCGA-LUSC sample, ESTIMATE to estimate the stroma and immunity levels of the samples, and obtained the methylation levels of CpG sites in TCGA-LUSC samples from the UCSC Xena platform. In conclusion, miR-205-5p and PTPRM interact with DNA methylation, tumor immunity and inflammation in the tumor microenvironment. MiR-205-5p and PTPRM had no correlation with TMB (Figure 6C).

## Discussion

The functional pattern of the miRNA-mRNA regulatory network has been shown in the occurrence and progression of various human diseases, including cancer (Muller and Nowak 2014; Wang et al., 2017; Cheng et al., 2018; Chen et al., 2020). In the current work, we aim to construct a potential miRNA-mRNA regulatory network in LUSC. Firstly, we researched the GEO database to screen the DE-miRNAs and DE-mRNAs preliminarily. The DE-miRNAs and DE-mRNAs showed consistent differential expression in 5 miRNA and 10 mRNA datasets. Then, the DE-miRNAs and DE-mRNAs preliminarily screened from GEO were further verified by TCGA-LUSC, miRcancer, and dbDEMC databases. Ultimately, 7 De-miRNAs (3 upregulated and 4 downregulated) and 270 DE-mRNAs (101 upregulated and 169 downregulated) showed consistent differential expression across the four databases. The miRNA-mRNA regulatory networks were verified by experimental screening from miRTarBase and Tarbase database, and 5 miRNA-mRNA regulatory networks were screened out. We used TCGA-LUSC data to conduct correlation analysis on the regulatory networks initially screened, and finally identified 4 miRNA-mRNA regulatory networks.

We know that the DE-miRNAs are involved in various pathways related to fatty acid metabolism and degradation. Fatty acid metabolism and fatty acid degradation have recently been recognized as an essential aberration of metabolism required for carcinogenesis (Swierczynski, Hebanowska, and Sledzinski 2014). Energy metabolism reprogramming, which fuels fast cell growth and proliferation by adjustments of energy metabolism, has been considered as an emerging hallmark of cancer (Hanahan and Weinberg 2011). In cancer cells, the biosynthesis of fatty acids often increases to meet the needs of lipid synthesis membranes and signaling molecules. Cancer cells usually obtain higher lipid accumulation in lipid droplets than normal cells (Catalina-Rodriguez et al., 2012).

After a series of bioinformatics analyses and external experimental verification, upregulated miR-205-5p and downregulated PTPRM in tumor tissues were finally screened out. The expression of miR-205-5p is closely associated with the incidence and development of tumors, such as head and neck cancer, ovarian cancer and breast cancer (Iorio et al., 2007; Tran et al., 2007; Wu and Mo 2009). It has been reported that the expression of miR-205 in non-small cell lung cancer (NSCLC) tissues is significantly increased and is related to the degree of tumor differentiation, which may lead to increased proliferation and invasion of lung cancer cells, thus leading to cancer progression (Lebanony et al., 2009; Jiang et al., 2013; Duan et al., 2017; Jiang et al., 2017). PTPRM is involved in cell-cell adhesion through same-sex interactions and may play a key role in signal transduction and growth regulation (Aricescu et al., 2006). PTPRM is associated with the prognosis of cervical cancer and promotes tumor growth and lymph node metastasis (Liu et al., 2020).

In this study, a panel combined with the network was built using logistic regression analysis. The expression of miR-205-5p and PTPRM has a good diagnostic efficacy in distinguishing LUSC patients from normal controls. The correlation analysis results of miR-205-5p and PTPRM with ssGSEA and tumor-associated phenotypes showed that miR-205-5p and PTPRM seemed to have a certain correlation with tumor immunity.

Tumor-infiltrating immune cells, such as T cells, macrophages, and neutrophils, are critical elements in tumor microenvironment and have shown close association with the clinical outcomes of various cancers (Gonzalez, Hagerling, and Werb 2018). T cells are composed of different subtypes with complicated phenotypes and functions, and tumor-infiltrating T cells play an extremely important role in the immune response system. Thymic epithelial cells (TECs) are essential regulators of T cell development and selection. miR-205-5p inhibits thymic epithelial cell proliferation via FA2H-TFAP2A feedback regulation in age-associated thymus involution (Gong et al., 2020). In glioma cells, miR-205-5p regulates TGF-β1 by targeting SMAD2, thereby influencing the immune response of TGF-β1 in tumors (Flavell et al., 2010; Meulmeester and Ten Dijke 2011; Duan and Chen 2016). Smad1 mediates BMP signaling and is involved in cell growth, apoptosis, morphogenesis and immune response (Bharathy et al., 2008). In esophageal squamous cell carcinoma, Mir-205-5p affects tumor immune response by targeting SMAD1 (Liang et al., 2014). PTPRM can activate STAT3 signaling pathway to enhance the anti-cancer immune responses and rescuing the suppressed immunologic microenvironment in tumors (Wang et al., 2018; Im et al., 2020).

CpG sites were identified in a region immediately upstream of the first exon of MIR205HG and in the miR-205 locus (Ferrari and Gandellini 2020). DNA methylation maintains the silencing of miR-205, thus playing a role in the occurrence and development of lung cancer (Tellez et al., 2011). The upregulation of FN1 reduced PTPRM by increasing its methylation, resulting in an increase of STAT3 phosphorylation and promoting GBM cell proliferation (Song et al., 2021). In general, miR-205-5p and PTPRM have a certain correlation with tumor immunity and global methylation, and we will further explore their role in tumor immunity in future experiments.

Although we have conducted a comprehensive analysis and experimental verification of the miRNA-mRNA regulatory networks involved in LUSC, the internal threats to the validity of this study be different platforms, study time, study population and experimental methods across different datasets, and limited sample size of external validation. Based on the above reasons, we selected DE-miRNAs or DE-mRNAs in each dataset to eliminate the influence caused by different datasets as much as possible. We will also expand the sample size and increase multi-center samples for further research in future experimental studies. External threats to the validity may require more experiments and clinical validation before they can be truly applied to clinical practice. In future research, we will try to cooperate with different centers and further evaluate the miRNA-mRNA regulatory networks based on larger sample sizes for more generalizable findings.

## Conclusion

In summary, we have constructed a miRNA-mRNA regulatory network that may be involved in the pathogenesis of LUSC. In the future, it is possible to help the treatment and prognosis of LUSC by targeting the established miRNA-mRNA regulatory network.

## Data Availability

The original contributions presented in the study are included in the article/Supplementary Material, further inquiries can be directed to the corresponding authors.

## Abbreviations

AUC, Area Under Curve; BH, Benjamini–Hochberg; CI, confidence interval; dbDEMC, database of Differentially Expressed MiRNAs in human Cancers; DCA, decision curve analysis; De-miRNAs, differential miRNAs; De-mRNAs, differential mRNAs; ESTIMATE, Estimation of STromal and Immune cells in MAlignant Tumour tissues using Expression data; GEO, gene expression omnibus; GEP, gene expression profiles; GO, Gene Ontology; GSEA, Single sample gene set Enrichment analysis; HR, hazard ratio; KEGG, Kyoto Encyclopedia of Genes Genomesss; LUSC, lung squamous cell carcinoma; miRNA, microRNA; NSCLC, non-small cell lung cancer; qRT-PCR, Real-Time Quantitative Reverse Transcription-Polymerase Chain Reaction; ROC, receiver operating characteristic curve; TCGA, the cancer genome atlas; TMB, tumor mutation burden.
