# Supplementary material for: Construction of the miRNA-mRNA Regulatory Networks and Explore Their Role in the Development of Lung Squamous Cell Carcinoma
Source: Front Mol Biosci. 2022 May 31;9:888020. doi: 10.3389/fmolb.2022.888020 (PMC9197544; doi:10.3389/fmolb.2022.888020)
Supplement: Supplementary file 1 [file DataSheet1.docx]

**Figure S1**

GO and KEGG pathway analysis show the associated function of DE-miRNAs and DE-mRNAs.

1. The KEGG of DE-miRNAs; (B)The GO of DE-miRNAs; (C)The KEGG of DE-mRNAs; (D)The GO of DE-mRNAs.


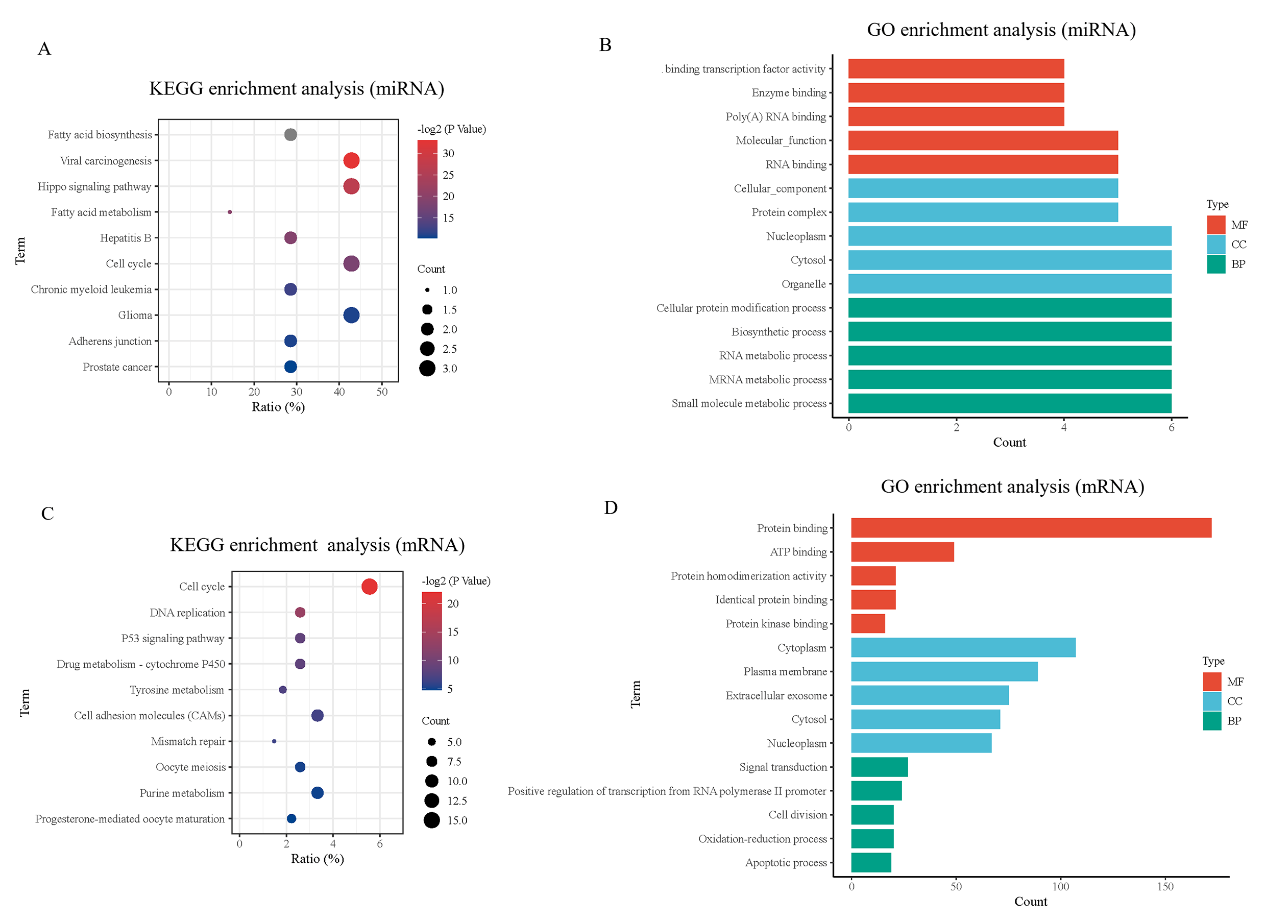


**Figure S2**

Immunohistochemistry images of PTPRM in LUSC and NC from HPA database.

(A): Medium immunostaining of PTPRM in normal lung alveolar cells (antibody CAB022443); (B)Immunostaining of PTPRM was not detected in lung squamous cell carcinoma cells (antibody CAB022443).


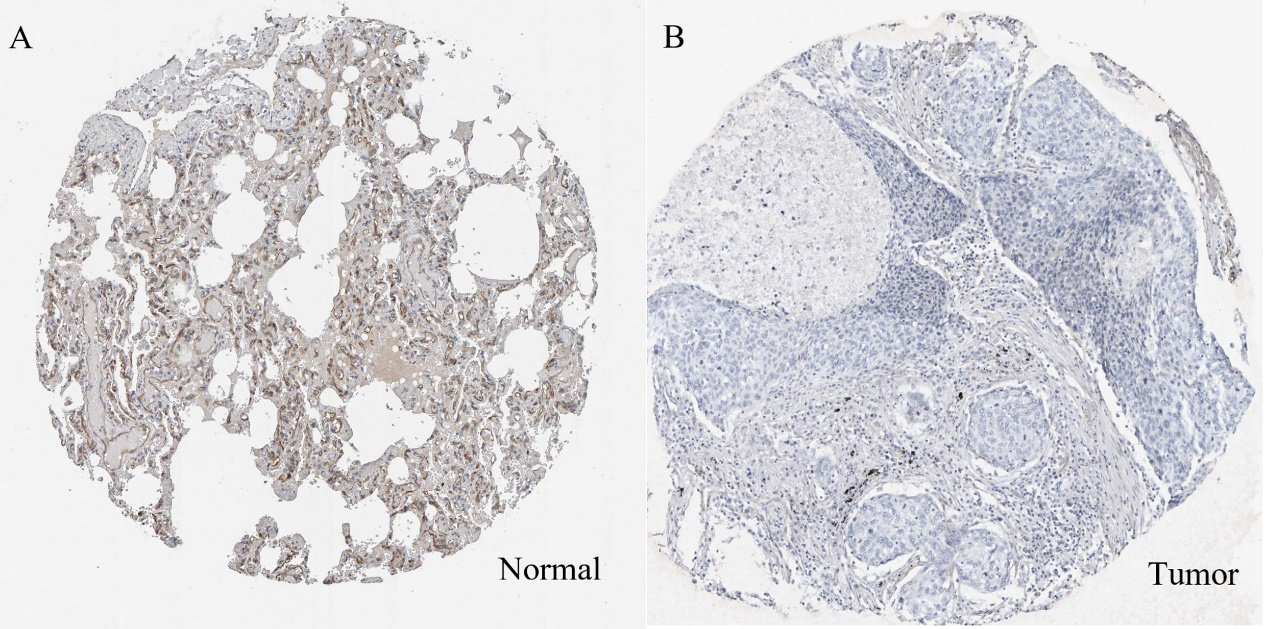


**Figure S3**

The expression of miR-205-5p and PTPRM in subgroups based on clinical pathological features of LUSC patients in TCGA. (Data are presented as mean±SEM; **p < 0.01)

1. The expression of miR-205-5p in stage I and stage II-IV (p=0.8410);
2. The expression of PTPRM in stage I versus stage II-IV (p=0.5950);
3. The expression of miR-205-5p in females and males (p=0.0073);
4. The expression of PTPRM in females and males (p=0.0947).;
5. The expression of miR-205-5p in age ≤68 and age >68 (p=0.171);
6. The expression of PTPRM in age ≤68 and age >68 (p=0.039).


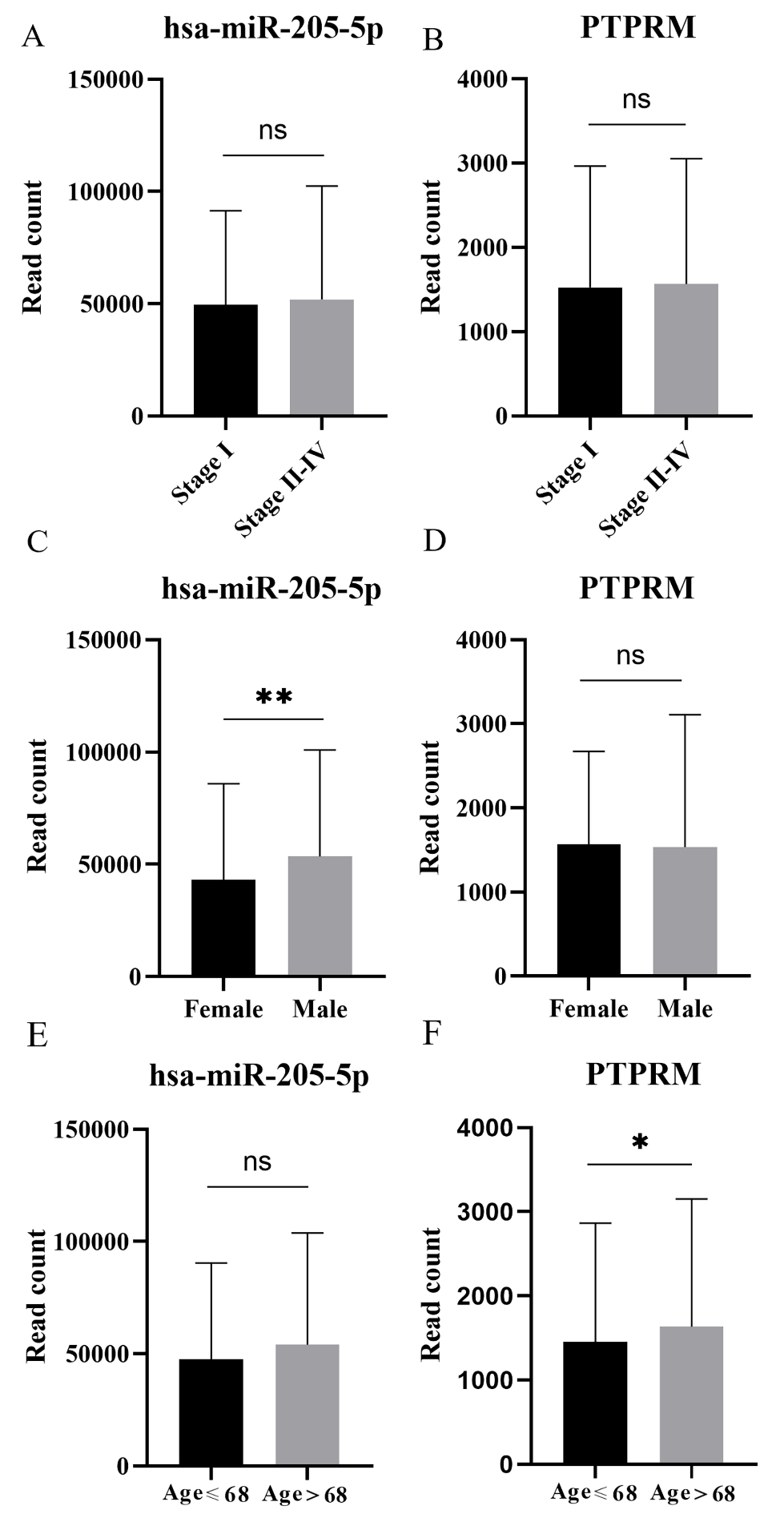


**Figure S4**

The survival analysis of miR-205-5p and PTPRM in TCGA-LUSC.

(A)has-miR-205-5p (p=0.21); (B)PTPRM (p=0.058).


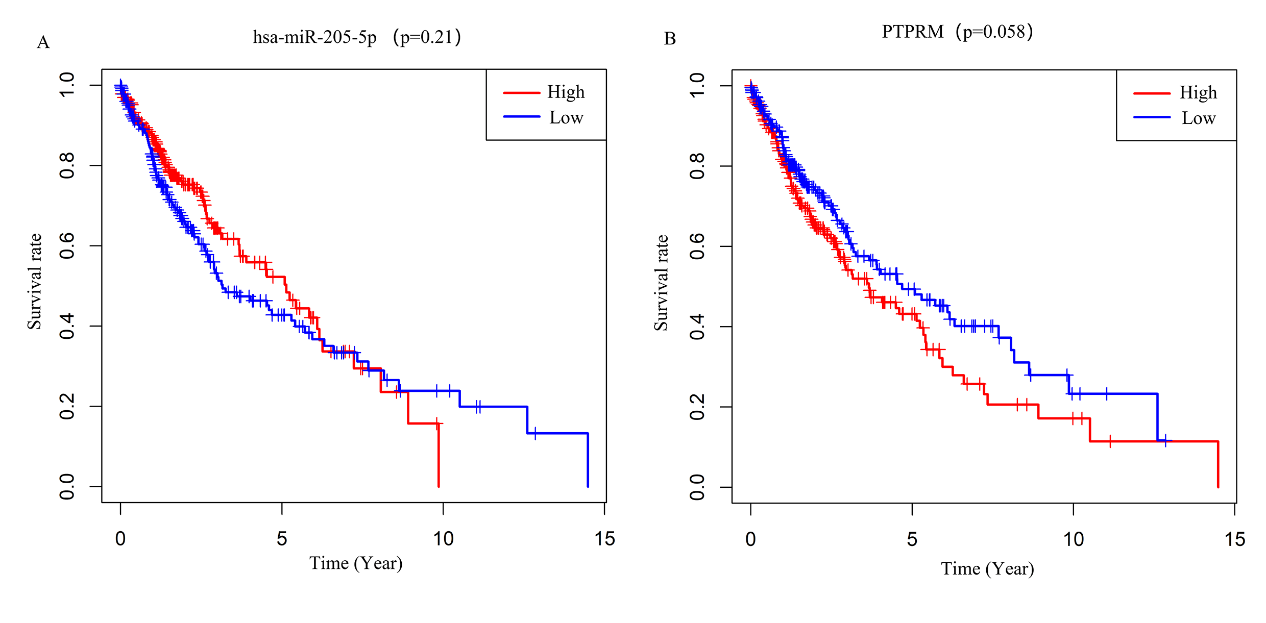


| **Table S1: The sequences of primers for candidate miRNAs and targeted mRNAs.** | | | |
| --- | --- | --- | --- |
|  | **Name** | **Forword Primer Sequences (5'→3')** | **Reverse Primer Sequences (5'→3')** |
| **miRNA mature** | hsa-miR-140-3p | TACCACAGGGTAGAACCA | universal reverse primer |
|  | hsa-miR-182-5p | TTTGGCAATGGTAGAACTCACA | universal reverse primer |
|  | hsa-miR-205-5p | TCCTTCATTCCACCGGAGT | universal reverse primer |
|  | hsa-miR-210-3p | CTGTGCGTGTGACAGCGGCT | universal reverse primer |
| **mRNA** | PTPRM | TCCAGCAAGAGTAATTCTCCTCC | CATGTACGTGTTGGGTCTCCA |
|  | GPD1L | ATCAAGGGCATAGACGAGGG | TCTGCATCATCAACCACGGTA |
|  | FOXF2 | AATGCCACTCGCCCTACAC | CGTTCTGGTGCAAGTAGCTCT |
|  | UBE2C | GACCTGAGGTATAAGCTCTCGC | CAGGGCAGACCACTTTTCCTT |
| **Reference gene** | RUN6B | CGATAAAATTGGAACGATACAGA | ATTTGGACCATTTCTCGATTTGT |
|  | 18s rRNA | CGCAGCTAGGAATAATGGAATAGG | GCCTCAGTTCCGAAAACCAA |

| **Table S2: The list of DE-miRNAs and DE-mRNAs (up-regulated or down-regulated in LUSC)** | | | |
| --- | --- | --- | --- |
| **DE-miRNA (down)** | **DE-mRNA(up)** | **DE-miRNA(up)** | **DE-mRNA(down)** |
| hsa-miR-125a-5p | ABCC5 | hsa-miR-210-3p | ABCA3 |
| hsa-miR-451a | ACTL6A | hsa-miR-182-5p | ABCA6 |
| hsa-miR-486-5p | ADAM12 | hsa-miR-205-5p | ABCA8 |
| hsa-miR-140-3p | ADAM23 |  | ABLIM3 |
|  | ATAD2 |  | ACADL |
|  | AURKA |  | ACVRL1 |
|  | BCL11A |  | ADAMTSL3 |
|  | BLM |  | ADARB1 |
|  | BMP7 |  | ADCY4 |
|  | BRCA1 |  | ADH1B |
|  | BUB1 |  | AGR3 |
|  | CASK |  | AK1 |
|  | CBX3 |  | AKAP12 |
|  | CCNA2 |  | AKAP13 |
|  | CCNB1 |  | ALDH3B1 |
|  | CCNB2 |  | ALOX5AP |
|  | CCT2 |  | ANGPT1 |
|  | CDC20 |  | ANXA11 |
|  | CDC6 |  | AOC3 |
|  | CDCA7 |  | AOX1 |
|  | CENPE |  | AQP1 |
|  | CENPF |  | AQP4 |
|  | CHAF1A |  | ARHGEF10 |
|  | CHAF1B |  | ARHGEF17 |
|  | CHEK1 |  | ARHGEF6 |
|  | CHEK2 |  | ARRB1 |
|  | CKAP2 |  | BTNL9 |
|  | CKS2 |  | C1orf116 |
|  | CRABP2 |  | C7 |
|  | CSE1L |  | CAB39L |
|  | CTHRC1 |  | CACNA2D2 |
|  | DLG5 |  | CADM1 |
|  | DNM1L |  | CAT |
|  | DSC3 |  | CAV1 |
|  | DSP |  | CCDC69 |
|  | DTL |  | CD34 |
|  | DUS4L |  | CD93 |
|  | EZH2 |  | CDKN1C |
|  | FKBP4 |  | CFD |
|  | FOXM1 |  | CLDN18 |
|  | GART |  | CLIC5 |
|  | GGH |  | CORO2B |
|  | GMPS |  | COX7A1 |
|  | H2AFZ |  | CRIP1 |
|  | HELLS |  | CRIP2 |
|  | HIST1H2BD |  | CSF3 |
|  | HMGB2 |  | CYB5A |
|  | HMMR |  | CYBRD1 |
|  | HPRT1 |  | CYP4B1 |
|  | IGFBP3 |  | DACH1 |
|  | KIAA0101 |  | DAPK1 |
|  | KIF11 |  | DAPK2 |
|  | KIF23 |  | DLC1 |
|  | KRT16 |  | DOCK4 |
|  | LYPD3 |  | DPT |
|  | MCM3 |  | EDNRA |
|  | MCM4 |  | EDNRB |
|  | MCM6 |  | EFEMP1 |
|  | MKI67 |  | EMCN |
|  | MMP12 |  | ENPP4 |
|  | MRPL13 |  | F8 |
|  | MSH2 |  | FABP4 |
|  | MTHFD2 |  | FAM107A |
|  | MTX2 |  | FBLN1 |
|  | MYBL2 |  | FBLN5 |
|  | NCAPG2 |  | FCER1A |
|  | NRCAM |  | FGFR4 |
|  | NTS |  | FHL1 |
|  | NUP155 |  | FMO2 |
|  | PAK1 |  | FMO5 |
|  | PCNA |  | FOLR1 |
|  | PMAIP1 |  | FOXF1 |
|  | POLB |  | FOXF2 |
|  | PRDX4 |  | GATA6 |
|  | PRKDC |  | GNG11 |
|  | PSMD14 |  | GPD1L |
|  | PTHLH |  | GPM6B |
|  | RAD51 |  | GPX3 |
|  | RAE1 |  | GRK5 |
|  | RFC4 |  | GSTM5 |
|  | RFC5 |  | GYPC |
|  | RNASEH2A |  | HLF |
|  | RRM2 |  | HPGD |
|  | SHFM1 |  | HSD17B6 |
|  | SHMT2 |  | HYAL1 |
|  | SLC2A1 |  | ID4 |
|  | SLC39A6 |  | IL6ST |
|  | SPP1 |  | ITGA8 |
|  | SRPK1 |  | ITM2A |
|  | STMN1 |  | JAM3 |
|  | SULF1 |  | KCNK3 |
|  | TFDP1 |  | KLF9 |
|  | TMEM106C |  | LIMCH1 |
|  | TOPBP1 |  | LMO2 |
|  | TPX2 |  | LMO3 |
|  | UBE2C |  | LMO7 |
|  | UCHL1 |  | LPL |
|  | VSNL1 |  | LTBP2 |
|  | WDHD1 |  | MAL |
|  | YEATS2 |  | MAMDC2 |
|  | ZWINT |  | MAOB |
|  |  |  | ME3 |
|  |  |  | MFAP4 |
|  |  |  | MGLL |
|  |  |  | MSLN |
|  |  |  | MUC1 |
|  |  |  | MYADM |
|  |  |  | MYH11 |
|  |  |  | NEDD9 |
|  |  |  | NFIB |
|  |  |  | NPR1 |
|  |  |  | NR3C2 |
|  |  |  | PDE8B |
|  |  |  | PDK4 |
|  |  |  | PDLIM3 |
|  |  |  | PDZRN3 |
|  |  |  | PIP5K1B |
|  |  |  | PKIG |
|  |  |  | PLLP |
|  |  |  | PODXL |
|  |  |  | PON3 |
|  |  |  | PRELP |
|  |  |  | PTPRM |
|  |  |  | RAMP3 |
|  |  |  | RBPMS |
|  |  |  | RGN |
|  |  |  | RGS5 |
|  |  |  | RHOBTB2 |
|  |  |  | ROR1 |
|  |  |  | RPS6KA2 |
|  |  |  | RRAD |
|  |  |  | S100A4 |
|  |  |  | SASH1 |
|  |  |  | SCNN1B |
|  |  |  | SDC4 |
|  |  |  | SELENBP1 |
|  |  |  | SELP |
|  |  |  | SLC16A5 |
|  |  |  | SLC1A1 |
|  |  |  | SLC39A8 |
|  |  |  | SLC4A4 |
|  |  |  | SLIT2 |
|  |  |  | SMAD7 |
|  |  |  | SOD3 |
|  |  |  | SORBS1 |
|  |  |  | SORBS2 |
|  |  |  | SPTBN1 |
|  |  |  | STARD13 |
|  |  |  | STX11 |
|  |  |  | STX2 |
|  |  |  | STXBP1 |
|  |  |  | SUSD2 |
|  |  |  | SVEP1 |
|  |  |  | TBX2 |
|  |  |  | TEK |
|  |  |  | TGFBR2 |
|  |  |  | TGFBR3 |
|  |  |  | TIE1 |
|  |  |  | TIMP3 |
|  |  |  | TLE2 |
|  |  |  | TMEM125 |
|  |  |  | TMEM47 |
|  |  |  | TRAK2 |
|  |  |  | TSPAN13 |
|  |  |  | VGLL3 |
|  |  |  | VSIG2 |
|  |  |  | VSIG4 |
|  |  |  | WFS1 |
|  |  |  | WIF1 |

**Table S3: The complete predicted network for miR-205-5p in TarBase and miRTarBase.**

| **Tarbase** | **miRTarBase** |
| --- | --- |
| BCL2 | BCL2 |
| CCNJ | CCNJ |
| CDK1 | CDK1 |
| CREB1 | CREB1 |
| CTGF | CTGF |
| CYR61 | CYR61 |
| E2F1 | E2F1 |
| E2F5 | E2F5 |
| ERBB3 | ERBB3 |
| ESRRG | ESRRG |
| HMGB3 | HMGB3 |
| HSPA8 | HSPA8 |
| KCNJ10 | KCNJ10 |
| LYN | LYN |
| MED1 | MED1 |
| MED13 | MED13 |
| NCAPG | NCAPG |
| NDUFA4 | NDUFA4 |
| PICALM | PICALM |
| PTEN | PTEN |
| PTPRJ | PTPRJ |
| PTPRM | PTPRM |
| RAN | RAN |
| RTN3 | RTN3 |
| SPDL1 | SPDL1 |
| SRC | SRC |
| TMSB4X | TMSB4X |
| VEGFA | VEGFA |
| YES1 | YES1 |
| YWHAH | YWHAH |
| ZEB1 | ZEB1 |
| AAGAB | ABCF1 |
| AAMP | ACP1 |
| AC004381.6 | ACSL1 |
| AC004925.1 | ACSL4 |
| ACAD8 | ACTRT3 |
| ACBD5 | AFF1 |
| ACD | AFF4 |
| ACP6 | AMOT |
| ACTR3 | ANGPTL7 |
| ACYP1 | AR |
| ADAT1 | B4GALT5 |
| ADORA2B | B4GALT6 |
| ADPGK | BAMBI |
| AGK | BCL6 |
| AGPAT6 | BCL9L |
| AHNAK | BDP1 |
| AICDA | BORCS5 |
| AIM1 | C11orf74 |
| AKAP9 | C1orf123 |
| AKT1 | C6orf201 |
| ALDH2 | CDK6 |
| ALDH9A1 | CDKN2AIPNL |
| ALG5 | CENPF |
| ALG9 | CFAP65 |
| ANAPC15 | CLIP1 |
| ANG | CPEB3 |
| ANKRD52 | CUL5 |
| ANP32E | DHCR24 |
| ANXA3 | DMXL2 |
| APOBEC3B | DNAJA1 |
| APTX | EGLN2 |
| AREG | EID2B |
| AREL1 | ENPP4 |
| ARHGAP19 | ERBB2 |
| ARHGAP29 | ETF1 |
| ARHGDIA | ETNK1 |
| ARPC5L | EZR |
| ASCC2 | F2RL2 |
| ASF1B | GM2A |
| ASNS | GNAS |
| ASPM | GOT1 |
| ATAD2 | GUCD1 |
| ATF3 | HMGB1 |
| ATF4 | HOXA11 |
| ATL3 | ICK |
| ATP2C2 | IL24 |
| ATP5C1 | IL32 |
| ATP9A | IMPAD1 |
| ATRX | INPPL1 |
| ATXN10 | IPO7 |
| AURKA | ITGA5 |
| AURKB | JMJD1C |
| AXL | KCTD16 |
| AZIN1 | KCTD20 |
| B3GNT1 | KLHL5 |
| B4GALT4 | LAMC1 |
| BAG6 | LCOR |
| BAIAP2L1 | LMNA |
| BCCIP | LPCAT1 |
| BDNF | LRP1 |
| BEX4 | LRRC59 |
| BICC1 | LRRK2 |
| BIRC3 | LRRTM4 |
| BIRC5 | LYSMD3 |
| BLCAP | MAML2 |
| BLVRA | MAOA |
| BNIP3L | MAP3K9 |
| BOD1 | MAPK14 |
| BRCA1 | MARCKS |
| BTG3 | MDH2 |
| BUB1B | MGLL |
| BUD31 | MMD |
| C11orf31 | MRPL44 |
| C12orf45 | NDUFB2 |
| C16orf59 | NEK9 |
| C19orf70 | NFAT5 |
| C20orf27 | NIPA2 |
| C21orf88 | NOTCH2 |
| C4orf27 | NOX5 |
| C5orf15 | NPRL3 |
| C5orf22 | NSF |
| C7orf55 | NUDT21 |
| C9orf78 | NUFIP2 |
| CACYBP | PAIP2B |
| CALM1 | PANK1 |
| CAMTA1 | PAPPA-AS1 |
| CAPZA2 | PARD6B |
| CARS | PHC2 |
| CBR3 | PHF12 |
| CBWD1 | PHF8 |
| CBWD5 | PHLPP2 |
| CBX1 | PIP4P1 |
| CBX6 | PLAGL2 |
| CCBL2 | PLCXD2 |
| CCDC15 | PRDX2 |
| CCDC53 | PRKCE |
| CCNA1 | PRLR |
| CCNA2 | PRR15 |
| CCNB1 | PRRG4 |
| CCNB2 | RAB11FIP1 |
| CCND1 | RAP2B |
| CCNE2 | RBBP4 |
| CCNG1 | RFX7 |
| CCPG1 | RGS6 |
| CDC123 | RNF217 |
| CDC20 | RUNX2 |
| CDC23 | SARAF |
| CDC25A | SATB2 |
| CDC25B | SERINC3 |
| CDC25C | SERTAD2 |
| CDC45 | SHISA6 |
| CDC6 | SLC25A25 |
| CDCA3 | SLC38A1 |
| CDCA8 | SLC39A14 |
| CDK2AP2 | SLC41A1 |
| CDKN2C | SLC5A12 |
| CDKN3 | SLC7A2 |
| CDT1 | SMAD1 |
| CDV3 | SMAD2 |
| CEBPB | SMAD4 |
| CEBPG | SMIM13 |
| CENPE | SMNDC1 |
| CENPM | SQLE |
| CENPU | SRD5A1 |
| CEP55 | STK38L |
| CEP57 | TAF11 |
| CFH | TCF20 |
| CHAC1 | TEX35 |
| CHAF1A | TM9SF2 |
| CHAF1B | TMEM123 |
| CHCHD3 | TMEM201 |
| CHEK1 | TMEM239 |
| CHEK2 | TNFSF8 |
| CHMP1B | TOLLIP |
| CHMP2A | TP73 |
| CHMP2B | TRAF3IP1 |
| CHMP5 | TXNL1 |
| CHN1 | UBE2Z |
| CHURC1 | UVRAG |
| CIRBP | VAMP1 |
| CKLF | VPS52 |
| CKS1B | VTI1A |
| CKS2 | XPOT |
| CLDN1 | XPR1 |
| CLN6 | YEATS2 |
| CLSTN1 | YY1 |
| CLTC | ZEB2 |
| CMKLR1 | ZNF585B |
| COBLL1 | ZSWIM5 |
| COPS5 |  |
| CPE |  |
| CRELD2 |  |
| CRLF3 |  |
| CRYZ |  |
| CSNK1A1 |  |
| CTSC |  |
| CTSO |  |
| CTSV |  |
| CTTN |  |
| CUZD1 |  |
| CXCL1 |  |
| CYB5A |  |
| CYTH2 |  |
| DCAF13 |  |
| DCAF6 |  |
| DCBLD2 |  |
| DCPS |  |
| DDB1 |  |
| DDIT3 |  |
| DDX47 |  |
| DENND1A |  |
| DENR |  |
| DEPDC1 |  |
| DESI2 |  |
| DEXI |  |
| DHRS3 |  |
| DIO3 |  |
| DLG4 |  |
| DLGAP5 |  |
| DMBT1 |  |
| DNAJB9 |  |
| DNAJC13 |  |
| DNAJC7 |  |
| DNAJC9 |  |
| DNM1L |  |
| DNMT1 |  |
| DONSON |  |
| DPM1 |  |
| DRAM1 |  |
| DSN1 |  |
| DTL |  |
| DUS1L |  |
| DUSP14 |  |
| DUSP22 |  |
| DYNLRB1 |  |
| ECSIT |  |
| ECT2 |  |
| EED |  |
| EIF3B |  |
| EIF3E |  |
| EIF4A1 |  |
| EIF4E2 |  |
| EIF4EBP1 |  |
| ELMO2 |  |
| ELOVL5 |  |
| ENTPD3 |  |
| EPCAM |  |
| EPRS |  |
| ERCC6L |  |
| ESPL1 |  |
| ESRP1 |  |
| EWSR1 |  |
| EXO1 |  |
| EXOSC8 |  |
| EZH2 |  |
| F2RL1 |  |
| FAM102A |  |
| FAM129A |  |
| FAM136A |  |
| FAM63B |  |
| FAM64A |  |
| FANCG |  |
| FANCI |  |
| FBN1 |  |
| FBXO5 |  |
| FEM1B |  |
| FEN1 |  |
| FERMT2 |  |
| FGF13 |  |
| FGFR1 |  |
| FKBP1A |  |
| FKBP9 |  |
| FN3KRP |  |
| FOPNL |  |
| FOS |  |
| FOXM1 |  |
| FSHR |  |
| FXN |  |
| GABARAPL1 |  |
| GABRD |  |
| GANAB |  |
| GAR1 |  |
| GARS |  |
| GBP2 |  |
| GCHFR |  |
| GGCT |  |
| GGH |  |
| GGNBP2 |  |
| GINS1 |  |
| GINS2 |  |
| GINS4 |  |
| GIPC1 |  |
| GLIS3 |  |
| GLOD4 |  |
| GLRX5 |  |
| GMNN |  |
| GPNMB |  |
| GPRC5A |  |
| GPSM2 |  |
| GRB10 |  |
| GRSF1 |  |
| GTF2A2 |  |
| GTF2H1 |  |
| GTF3A |  |
| GTF3C6 |  |
| GTPBP2 |  |
| GXYLT1 |  |
| HABP4 |  |
| HAX1 |  |
| HCAR2 |  |
| HECTD3 |  |
| HELLS |  |
| HIBCH |  |
| HINT2 |  |
| HJURP |  |
| HMGB2 |  |
| HMMR |  |
| HMOX1 |  |
| HNRNPA0 |  |
| HNRNPH3 |  |
| HOPX |  |
| HOXC8 |  |
| HRAS |  |
| HRSP12 |  |
| HSD17B7 |  |
| HSPA13 |  |
| HSPA9 |  |
| IDH2 |  |
| IER5 |  |
| IFI27L1 |  |
| IFI35 |  |
| IGBP1 |  |
| IL11 |  |
| IL17RB |  |
| IL6 |  |
| INO80C |  |
| INSIG1 |  |
| INSIG2 |  |
| IPO5 |  |
| IRF4 |  |
| IRGC |  |
| ITPK1 |  |
| IVNS1ABP |  |
| JUP |  |
| KAT2B |  |
| KCMF1 |  |
| KCNT2 |  |
| KDELR3 |  |
| KEAP1 |  |
| KIAA0430 |  |
| KIF11 |  |
| KIF13B |  |
| KIF14 |  |
| KIF15 |  |
| KIF18B |  |
| KIF20A |  |
| KIF2C |  |
| KIF4A |  |
| KIFAP3 |  |
| KIFC1 |  |
| KLC1 |  |
| KLF11 |  |
| KLHDC10 |  |
| KLHL2 |  |
| KLK2 |  |
| KNTC1 |  |
| KPNA2 |  |
| KRT16 |  |
| LARP6 |  |
| LEPREL1 |  |
| LETM2 |  |
| LGALS3BP |  |
| LHX2 |  |
| LIG1 |  |
| LIPH |  |
| LIPK |  |
| LONP1 |  |
| LPAR4 |  |
| LRIG1 |  |
| LRP10 |  |
| LRP3 |  |
| LRPAP1 |  |
| LRRC8D |  |
| LSM2 |  |
| LSM5 |  |
| LSM6 |  |
| LTA4H |  |
| LTF |  |
| LYPD6B |  |
| LYPLA2 |  |
| MAD1L1 |  |
| MALL |  |
| MAP2K1 |  |
| MAP7D1 |  |
| MAPRE3 |  |
| MAZ |  |
| MCM10 |  |
| MCM2 |  |
| MCM3 |  |
| MCM5 |  |
| MCM6 |  |
| MCM7 |  |
| MDC1 |  |
| MELK |  |
| METTL5 |  |
| MGAT4A |  |
| MGAT4B |  |
| MICAL2 |  |
| MICALL1 |  |
| MIS18A |  |
| MKI67 |  |
| MKNK2 |  |
| MLST8 |  |
| MNAT1 |  |
| MNS1 |  |
| MOCOS |  |
| MOXD1 |  |
| MRPL23 |  |
| MRPL4 |  |
| MRPL47 |  |
| MRPS14 |  |
| MRPS17 |  |
| MRPS23 |  |
| MRPS33 |  |
| MRPS7 |  |
| MT1B |  |
| MT1F |  |
| MT1H |  |
| MT1HL1 |  |
| MT1X |  |
| MT2A |  |
| MTA2 |  |
| MTHFD2 |  |
| MTIF2 |  |
| MTIF3 |  |
| MVK |  |
| MYBL1 |  |
| MYBL2 |  |
| MYL6 |  |
| MYO19 |  |
| NA |  |
| NABP2 |  |
| NAE1 |  |
| NCAPD2 |  |
| NCAPG2 |  |
| NCAPH |  |
| NCOA4 |  |
| NCOR2 |  |
| NDC1 |  |
| NDC80 |  |
| NDRG3 |  |
| NDST1 |  |
| NDUFA10 |  |
| NDUFA5 |  |
| NECAP1 |  |
| NEFH |  |
| NEIL3 |  |
| NELFB |  |
| NEU1 |  |
| NFKB1 |  |
| NGDN |  |
| NMU |  |
| NNMT |  |
| NOS3 |  |
| NPEPPS |  |
| NPTN |  |
| NQO1 |  |
| NR4A2 |  |
| NRN1 |  |
| NT5C |  |
| NT5DC2 |  |
| NUCB2 |  |
| NUDT1 |  |
| NUP37 |  |
| NUP62CL |  |
| NUPR1 |  |
| NUSAP1 |  |
| NXF1 |  |
| OCIAD2 |  |
| OIP5 |  |
| OPTN |  |
| OR13C4 |  |
| OR2M5 |  |
| ORC6 |  |
| ORMDL1 |  |
| OSBPL1A |  |
| PABPC4 |  |
| PAK2 |  |
| PAM |  |
| PARK7 |  |
| PARL |  |
| PARP2 |  |
| PARPBP |  |
| PBK |  |
| PCBD1 |  |
| PCDHA6 |  |
| PCK2 |  |
| PCNA |  |
| PDCL |  |
| PDE5A |  |
| PDE6C |  |
| PDLIM1 |  |
| PDRG1 |  |
| PEA15 |  |
| PFN2 |  |
| PHF10 |  |
| PHF11 |  |
| PHF5A |  |
| PHGDH |  |
| PHLDB2 |  |
| PIGC |  |
| PIH1D1 |  |
| PJA1 |  |
| PKMYT1 |  |
| PKN1 |  |
| PLA2G16 |  |
| PLK1 |  |
| PLK2 |  |
| PLXDC2 |  |
| PNMA1 |  |
| PODXL |  |
| POLA2 |  |
| POLD3 |  |
| POLE2 |  |
| POLE4 |  |
| POLQ |  |
| POLR2B |  |
| POLR2I |  |
| PRC1 |  |
| PRELID1 |  |
| PREP |  |
| PRIM1 |  |
| PRKRIR |  |
| PRMT1 |  |
| PRPS1 |  |
| PRSS23 |  |
| PSAP |  |
| PSAT1 |  |
| PSMB8 |  |
| PSMD1 |  |
| PSMD13 |  |
| PSMD2 |  |
| PSMD7 |  |
| PSMD8 |  |
| PSME2 |  |
| PSMG1 |  |
| PSMG4 |  |
| PSRC1 |  |
| PTP4A2 |  |
| PTPN20A |  |
| PTPRK |  |
| PTPRR |  |
| PTRF |  |
| PTTG1 |  |
| PWP1 |  |
| PWP2 |  |
| PYGB |  |
| R3HCC1 |  |
| RAB11A |  |
| RAB15 |  |
| RAB1A |  |
| RAB31 |  |
| RAB35 |  |
| RAB5C |  |
| RAB8A |  |
| RACGAP1 |  |
| RAD21 |  |
| RAD51 |  |
| RAD51AP1 |  |
| RAD51C |  |
| RARS |  |
| RASSF1 |  |
| RBBP8 |  |
| RBM17 |  |
| RBX1 |  |
| RECQL4 |  |
| REEP4 |  |
| RELN |  |
| RFC2 |  |
| RFC4 |  |
| RFC5 |  |
| RFXANK |  |
| RMI1 |  |
| RNASEH2A |  |
| RNF114 |  |
| RNF144A |  |
| RNF167 |  |
| RNF168 |  |
| RNF169 |  |
| RNF170 |  |
| RNF181 |  |
| RNF182 |  |
| RNF220 |  |
| ROCK1 |  |
| RPA2 |  |
| RPA3 |  |
| RPF2 |  |
| RPL15 |  |
| RPL26L1 |  |
| RPL37A |  |
| RPRD1B |  |
| RPS19BP1 |  |
| RPS6KB2 |  |
| RRAGA |  |
| RRAGD |  |
| RRM1 |  |
| RRM2 |  |
| S100P |  |
| SARS |  |
| SAV1 |  |
| SCML2 |  |
| SCN5A |  |
| SCRN1 |  |
| SDC4 |  |
| SDHB |  |
| SEC11C |  |
| SEH1L |  |
| SEMA7A |  |
| SENP2 |  |
| SEPHS2 |  |
| SERPINA3 |  |
| SERPIND1 |  |
| SERPINE1 |  |
| SERPINE2 |  |
| SETD3 |  |
| SFXN4 |  |
| SH3PXD2B |  |
| SHCBP1 |  |
| SHIP2(hsa) |  |
| SHISA2 |  |
| SHMT2 |  |
| SIAH2 |  |
| SKP2 |  |
| SLC12A1 |  |
| SLC12A7 |  |
| SLC14A1 |  |
| SLC1A4 |  |
| SLC1A5 |  |
| SLC25A1 |  |
| SLC25A15 |  |
| SLC25A4 |  |
| SLC31A1 |  |
| SLC3A2 |  |
| SLC4A4 |  |
| SLC6A9 |  |
| SLC7A11 |  |
| SLMO2 |  |
| SMC2 |  |
| SMC4 |  |
| SMCO4 |  |
| SMPD4 |  |
| SMTN |  |
| SMYD2 |  |
| SNAI2 |  |
| SNW1 |  |
| SP4 |  |
| SPAG1 |  |
| SPAG5 |  |
| SPC25 |  |
| SPG11 |  |
| SPTBN1 |  |
| SQSTM1 |  |
| SRPR |  |
| STC2 |  |
| STIL |  |
| STMN1 |  |
| STOML2 |  |
| STX6 |  |
| STXBP3 |  |
| SULT1A2 |  |
| SUMO3 |  |
| SUPT16H |  |
| SYNPR |  |
| TACC3 |  |
| TAPBPL |  |
| TARBP2 |  |
| TAS2R39 |  |
| TBC1D7 |  |
| TBCE |  |
| TCEA1 |  |
| TCEAL4 |  |
| TDG |  |
| TDP1 |  |
| TDRD7 |  |
| TERF2IP |  |
| TFAP2C |  |
| TFG |  |
| THAP10 |  |
| THBS1 |  |
| THYN1 |  |
| TIAL1 |  |
| TIMM10 |  |
| TIMM8A |  |
| TIMM9 |  |
| TIMP2 |  |
| TIPIN |  |
| TIPRL |  |
| TK1 |  |
| TMCO3 |  |
| TMEM106C |  |
| TMEM110 |  |
| TMEM126A |  |
| TMEM140 |  |
| TMEM2 |  |
| TMEM39B |  |
| TMEM41B |  |
| TMEM51 |  |
| TMPRSS2 |  |
| TNFAIP1 |  |
| TNFAIP8 |  |
| TNIP2 |  |
| TOMM22 |  |
| TOP2A |  |
| TOR1AIP2 |  |
| TP53 |  |
| TP53BP1 |  |
| TPD52L1 |  |
| TPX2 |  |
| TRA2B |  |
| TRAIP |  |
| TRAK2 |  |
| TRAM1 |  |
| TRAPPC2L |  |
| TRIB1 |  |
| TRIB3 |  |
| TRIM16 |  |
| TRIP13 |  |
| TROAP |  |
| TSPYL4 |  |
| TTC3 |  |
| TTK |  |
| TTLL7 |  |
| TUBB |  |
| TUSC1 |  |
| UBE2C |  |
| UBE2D3 |  |
| UBE2J2 |  |
| UBE2S |  |
| UBE2T |  |
| UBXN1 |  |
| UCHL3 |  |
| UGP2 |  |
| UPP1 |  |
| VASN |  |
| VBP1 |  |
| VDAC2 |  |
| VIM |  |
| VPS16 |  |
| VPS37C |  |
| VTI1B |  |
| WARS |  |
| WDR49 |  |
| WEE1 |  |
| WIPI1 |  |
| WRAP53 |  |
| WSB2 |  |
| XBP1 |  |
| XCL2 |  |
| XPC |  |
| XPO5 |  |
| XYLT2 |  |
| YAP1 |  |
| YKT6 |  |
| YLPM1 |  |
| YPEL1 |  |
| YRDC |  |
| YTHDF1 |  |
| YWHAB |  |
| ZBED1 |  |
| ZFAND3 |  |
| ZMYM2 |  |
| ZNF212 |  |
| ZNF382 |  |
| ZNF428 |  |
| ZNF48 |  |
| ZNF638 |  |
| ZNF645 |  |
| ZNF827 |  |
| ZWINT |  |
| ZXDC |  |

| **Table S4：Pearson's correlation analysis of miRNA-mRNA networks in TCGA-LUSC.** | | | |
| --- | --- | --- | --- |
| ***miRNA (up)*** | ***mRNA (down)*** | ***p-value*** | ***r-value*** |
| 210-3p | GPD1L | **0.0099** | **-0.1189** |
| 182-5p | FOXF2 | **0.0151** | **-0.1120** |
|  | PDK4 | 0.2599 | -0.0521 |
| 205-5p | PTPRM | **0.0001** | **-0.1748** |
| ***miRNA (down)*** | ***mRNA (up)*** | ***p-value*** | ***r-value*** |
| 140-3p | UBE2C | **0.0000** | **-0.2157** |

| **Table S5: Immune cells differentiated between tumor tissue and normal tissue in TCGA-LUSC data.** | | | | |
| --- | --- | --- | --- | --- |
| **Cell type** | **Tumor** | **Normal** | **logFC** | ***P* Value** |
| Monocytes | 0.0055 | 0.0598 | -3.444 | 1.7868E-33 |
| Eosinophils | 0.0017 | 0.0099 | -2.500 | 3.2161E-12 |
| Neutrophils | 0.0126 | 0.0381 | -1.591 | 2.0681E-13 |
| NK cells resting | 0.0150 | 0.0388 | -1.370 | 4.3514E-10 |
| Mast cells resting | 0.0354 | 0.0648 | -0.872 | 5.6088E-06 |
| Dendritic cells activated | 0.0193 | 0.0332 | -0.784 | 3.2006E-05 |
| T cells CD4 memory resting | 0.1292 | 0.1988 | -0.622 | 2.8945E-08 |
| Macrophages M1 | 0.0798 | 0.0347 | 1.202 | 2.6148E-12 |
| Dendritic cells resting | 0.0335 | 0.0105 | 1.681 | 4.8061E-05 |
| T cells CD4 memory activated | 0.0309 | 0.0083 | 1.891 | 1.9944E-05 |
| Plasma cells | 0.0973 | 0.0173 | 2.488 | 9.4655E-17 |
| T cells follicular helper | 0.0309 | 0.0041 | 2.905 | 8.5558E-15 |
| T cells regulatory (Tregs) | 0.0198 | 0.0017 | 3.540 | 1.5359E-13 |
